# Allergic rhinitis and asthma symptoms in a real-life study of MP-AzeFlu to treat multimorbid allergic rhinitis and asthma

**DOI:** 10.1186/s12948-020-00130-9

**Published:** 2020-08-06

**Authors:** David Price, Ludger Klimek, Gabriella Gálffy, Melanie Emmeluth, Arkady Koltun, Ferdinand Kopietz, Duc Tung Nguyen, Ranny van Weissenbruch, Wolfgang Pohl, Hans-Christian Kuhl, Glenis Scadding, Joaquim Mullol

**Affiliations:** 1 Centre of Academic Primary Care, Division of Applied Health Sciences, University of Aberdeen, Aberdeen, AB25 2ZD UK; 2 Optimum Patient Care, Cambridge, UK; 3 Observational and Pragmatic Research Institute (OPRI), Pte | #02-05 883 North Bridge Road, Singapore, 198785 Singapore; 4 Zentrum für Rhinologie und Allergologie, Wiesbaden, Germany; 5 Pulmonology Hospital, Törökbálint, Hungary; 6 MEDA Pharma GmbH & Co. KG (A Mylan Company), Bad Homburg, Germany; 7 Mylan, Inc, Canonsburg, PA USA; 8 Wilhelmina Ziekenhuis Assen, Assen, The Netherlands; 9 Karl Landsteiner Gesellschaft, Institut für Klinische und experimentelle Pneumologie, Vienna, Austria; 10 Royal National Ear, Nose Throat Hospital, London, England; 11 Rhinology Unit & Smell Clinic, ENT Department, Hospital Clínic Barcelona, IDIBAPS, Universitat de Barcelona, CIBERES, Barcelona, Catalonia Spain

**Keywords:** Allergic rhinitis, Asthma, Comorbidity, Daily activity, MP-AzeFlu, Sleep quality, Visual analog scale

## Abstract

**Background:**

Asthma affects up to nearly 40% of patients with allergic rhinitis (AR). Poor control of AR symptoms is associated with poor asthma control. The goal of this study was to evaluate the effect of AR treatment with MP-AzeFlu on symptoms of AR as well as symptoms of asthma.

**Methods:**

This prospective study used a visual analog scale (VAS) to assess symptoms of AR and asthma before and after treatment with MP-AzeFlu (Dymista^®^; azelastine hydrochloride plus fluticasone propionate; 1 spray in each nostril twice daily for 2 weeks). Participants suffered from moderate-to-severe AR according to Allergic Rhinitis and its Impact on Asthma criteria, with acute AR symptoms (AR-VAS scores ≥ 50 mm) on inclusion day. In addition to symptom assessment, patients recorded the impact of AR symptoms on quality-of-life measures before, during, and at the conclusion of the treatment period (approximately 14 days). Patients self-reported change in frequency of their usage of asthma reliever medication on the last day of treatment.

**Results:**

Of 1103 study participants, 267 (24.2%) had comorbid asthma. These participants reported using a mean of 5.1 puffs of asthma reliever medication in the week before treatment with MP-AzeFlu. A total of 81.8% of patients with comorbid asthma responded to AR therapy (AR-VAS < 50 mm on at least 1 study day). Among patients with AR and comorbid asthma, MP-AzeFlu was associated with improved VAS scores across all study parameters, including AR symptom severity, asthma symptom severity, sleep quality, daily work or school activities, daily social activities, and daily outdoor activities. Asthma symptom severity decreased from a mean of 48.9 mm to 24.1 mm on the VAS. Self-reported frequency of asthma reliever medication use was reduced for 57.6% of participants (n = 139/241).

**Conclusion:**

MP-AzeFlu used to relieve AR symptoms was associated with reduced asthma symptom VAS scores and frequency of asthma reliever medication usage. Changes in overall symptoms of AR and asthma were correlated.

## Background

Globally, allergic rhinitis (AR) is a common, systemic allergic disease, with a prevalence of up to 25% in children and 40% in adults [1]. Among patients with AR, other allergic disorders are frequently comorbid [2]. Between 15% and nearly 40% of patients with AR have comorbid asthma, whereas asthma prevalence in the general population is approximately 7% [1, 3].

Among patients with AR who visited a general practitioner, the majority—more than 90%—have moderate-to-severe intermittent or persistent disease [4]. Many patients with moderate-to-severe AR have poorly controlled asthma [3], which may be attributable in part to lower airway inflammation [3]. In a survey of 520 patients with asthma, asthma was significantly less likely to be controlled in patients with moderate-to-severe, persistent AR compared with those with intermittent AR (65.7% vs 20.4%; *P* < 0.01) [4]. Furthermore, patients with AR and asthma comorbidity have higher healthcare resource utilization, including clinic visits, hospitalizations, and pharmacy costs over a 12-month time period [5]. Direct costs of AR are significantly higher for patients with mild-persistent asthma (€719) or moderate-persistent asthma (€799) than for the general population with AR (€554) [6].

Treatment of AR may concurrently improve AR and asthma symptoms in patients with comorbid disease [7–9]. In a past study, failure to manage AR symptoms was associated with increased use of asthma medications [7]. When patients with moderate-to-severe AR forgot to use their AR medication, more than half of those patients reported having to increase use of asthma reliever medications and 19.5% reported a need to increase asthma controller medication use [7]. Furthermore, in observational studies, AR treatment has been shown to improve upper and lower airway outcomes and decrease the risk for asthma-related hospitalization and emergency department visits by half [8, 9].

Treatments for AR include oral H_1_ antihistamines, intranasal corticosteroids (INCS), or intranasal antihistamines (INAH) [1]. Despite the wide variety of medication options available, many patients are dissatisfied with their AR treatment, resulting in poor adherence [10]. Therefore, combination therapies may improve satisfaction by reducing medication burden in patients with moderate-to-severe, persistent AR symptoms. In the Allergic Rhinitis and its Impact on Asthma (ARIA) 2016 guideline update, combination treatment with INCS and INAH is recommended for patients with AR [1].

Azelastine hydrochloride has been formulated with fluticasone propionate in a single intranasal spray (MP-AzeFlu; Dymista^®^) for the treatment of AR [11]. Compared with fluticasone propionate or azelastine hydrochloride alone, MP-AzeFlu resulted in significantly greater improvements in AR symptoms, including nasal congestion, one of the most bothersome and prevalent symptoms of AR, [12–14] nasal cell inflammation [15], loss of smell [16], and nasal hyperreactivity [17]. Relative to individual dosing of both an INAH and INCS, MP-AzeFlu was also associated with lower pharmacy costs and total costs in a prior database analysis [18].

The purpose of this analysis of a real-world study was to evaluate the effect of MP-AzeFlu on asthma symptoms and frequency of use of asthma reliever medication in patients with comorbid AR and asthma.

## Methods

### Study design

This was a multinational, multicenter, prospective, noninterventional, real-life study conducted in 6 European countries: Austria, Germany, Czech Republic, Hungary, Netherlands, and Ireland. The study ran from February 21, 2018, to April 30, 2019. Ethics approval was obtained according to guidelines and procedures of the respective countries. Physicians who were usually involved in the management of AR and routinely used a visual analog scale (VAS) for symptom assessment in patients with AR were invited to participate in the study. Participating physicians included general practitioners, allergists, otorhinolaryngologists, pulmonologists, dermatologists, and pediatricians.

The study consisted of an inclusion visit (day 0) and a control visit after about 14 days, allowing for some flexibility depending on usual clinical practice. Patients received patient cards at the inclusion visit to record AR symptoms, asthma symptoms, and other outcomes using a VAS. Physicians collected patient cards at the control visit, on or around day 14 or by mail.

### Participants

Physicians enrolled patients with moderate-to-severe seasonal or perennial AR according to ARIA criteria, for whom MP-AzeFlu was prescribed for the first time. Decisions to include patients in the study were made by the physicians independently from and after the decision to prescribe MP-AzeFlu to the patient.

Inclusion criteria included first-time prescription of MP-AzeFlu according to the summary of product characteristics, age 12 years or older, moderate-to-severe AR according to ARIA criteria [19], acute symptoms of AR on the day of inclusion (AR symptoms VAS ≥ 50 mm), written informed consent by the patient and (if applicable) caregiver for patients younger than 18 years, ability to understand the instructions for use of MP-AzeFlu according to the summary of product characteristics and patient leaflet, and ability to return the completed patient card.

Exclusion criteria included known allergic reactions to MP-AzeFlu or any of its ingredients, pregnancy or planned pregnancy, breastfeeding, inability to provide informed consent, or missing consent.

### Study treatment

All patients received MP-AzeFlu. MP-AzeFlu was dosed as outlined in the country-specific summary of product characteristics: 1 spray in each nostril twice daily (total daily dose: 548 µg azelastine hydrochloride and 200 µg fluticasone propionate) for 2 weeks. Physicians ensured that the patient properly understood the instructions for use, as specified in the summary of product characteristics and patient information leaflet.

### Study measures/outcomes

On day 0, the physician documented patient demographics, AR symptoms, and previous treatments of AR in an electronic case report form. Patient recollections of their AR symptoms over the past 24 h were measured using a printed single-line VAS (AR-VAS) in the patient card, ranging from “not at all bothersome” (0 mm) to “extremely bothersome” (100 mm). AR symptom severity VAS scores and, for patients who suffered from asthma, asthma symptom severity VAS scores, were documented on the patient’s card on days 0, 1, 3, 7, and ~ 14. Response was defined as an AR-VAS rating < 50 mm (indicating controlled AR) [20] at least once during the study.

On days 0, 7, and ~ 14, patients assessed their sleep quality and troublesomeness in daily activities over the past 7 days, from “not at all troubled” (0 mm) to “extremely troubled” (100 mm). For patients who suffered from asthma, information on frequency of use of asthma reliever medication was collected at baseline. At the end of the documentation period (day ~ 14), the self-reported change in the frequency of use of asthma reliever medication was recorded as significantly reduced, reduced, equal, increased, or significantly increased. All suspected adverse drug reactions were documented in the case reports.

### Statistical methods

Subpopulation analyses were performed for patients with AR but no asthma and for patients with AR and asthma comorbidity. The responder rate was calculated for the study population. Statistical analyses were performed using the statistical software package SAS (SAS Institute Inc.; Cary, NC, USA) version 9.4 or higher.

## Results

### Study population

Of 1154 enrolled patients, 51 were excluded from data analysis because their data had not been confirmed by the investigator. The 1103 remaining patients were included in the safety analysis. A total of 267 patients listed asthma as a comorbidity. Patient demographics and baseline characteristics are detailed in Table 1.

**Table 1:** Patient Baseline Demographics

Baseline characteristics	Total study population (N = 1103)	AR with no asthma (n = 836)	AR with asthma (n = 267)
Gender, n (%)			
Male	474 (43.0)	355 (42.5)	119 (44.6)
Female	624 (56.6)	478 (57.2)	146 (54.7)
Missing	5 (0.5)	3 (0.4)	2 (0.8)
Age, n (%)			
12–17 years	82 (7.4)	67 (8.0)	15 (5.6)
18–65 years	937 (85.0)	711 (85.0)	226 (84.6)
> 65 years	84 (7.6)	58 (6.9)	26 (9.7)
Allergic sensitization (number of allergens), n (%)			
1	178 (16.1)	152 (18.2)	26 (9.7)
2–5	570 (51.7)	428 (51.2)	142 (53.2)
> 5	176 (16.0)	96 (11.5)	80 (30.0)
Unknown	179 (16.2)	160 (19.1)	19 (7.1)
Type of AR, n (%)			
Perennial only	120 (10.9)	102 (12.2)	18 (6.7)
Seasonal only	435 (39.4)	354 (42.3)	81 (30.3)
Perennial and seasonal	444 (40.3)	285 (34.1)	159 (59.6)
Missing	104 (9.4)	95 (11.4)	9 (3.4)
Allergic comorbidities, n (%)			
Asthma	267 (24.2)	0 (0)	267 (100)
Dermatitis/eczema	127 (11.5)	90 (10.8)	37 (13.9)
Food allergy/allergies	109 (9.9)	81 (9.7)	28 (10.5)
Severe allergic reactions	30 (2.7)	18 (2.2)	12 (4.5)
None	593 (53.8)	593 (70.9)	0
Missing	89 (8.1)	89 (10.6)	0
Baseline AR-VAS scores, mean (SD)			
AR-VAS scores	73.2 (13.4)	72.8 (13.4)	74.1 (13.5)
Previous symptomatic AR treatments since last year, n (%)			
Oral, nonsedating H_1_-antihistamine	506 (45.9)		
Intranasal corticosteroid	471 (42.7)		
Intranasal decongestant	191 (17.3)		
Intranasal H_1_-antihistamine	177 (16.0)		
Oral, first-generation H_1_-antihistamine	162 (14.7)		
Ocular H_1_-antihistamine	133 (12.1)		
Oral or nebulized corticosteroid	99 (9.0)		
Intranasal mast cell stabilizer	62 (5.6)		
Oral leukotriene antagonist	50 (4.5)		
Ocular mast cell stabilizer	42 (3.8)		
Oral decongestant	26 (2.4)		
Other	54 (4.9)		
Unknown	24 (2.2)		
None	164 (14.9)		

*AR* indicates allergic rhinitis, *SD* standard deviation, *VAS* visual analog scale

^a^Percentages may not equal 100 due to rounding

### AR symptom response

In the total study population, all 1103 patients were included in the responder rate analysis. Among the 915 patients reporting previous AR treatment, the most commonly used symptomatic AR treatments were oral, nonsedating H_1_-antihistamine (n = 506; 45.9%), INCS (n = 471; 42.7%), intranasal decongestant (n = 191; 17.3%), INAH (n = 177; 16.0%), oral, first-generation H_1_-antihistamine (n = 162; 14.7%), and ocular H_1_-antihistamine (n = 133; 12.1%).

**Fig. 1:**
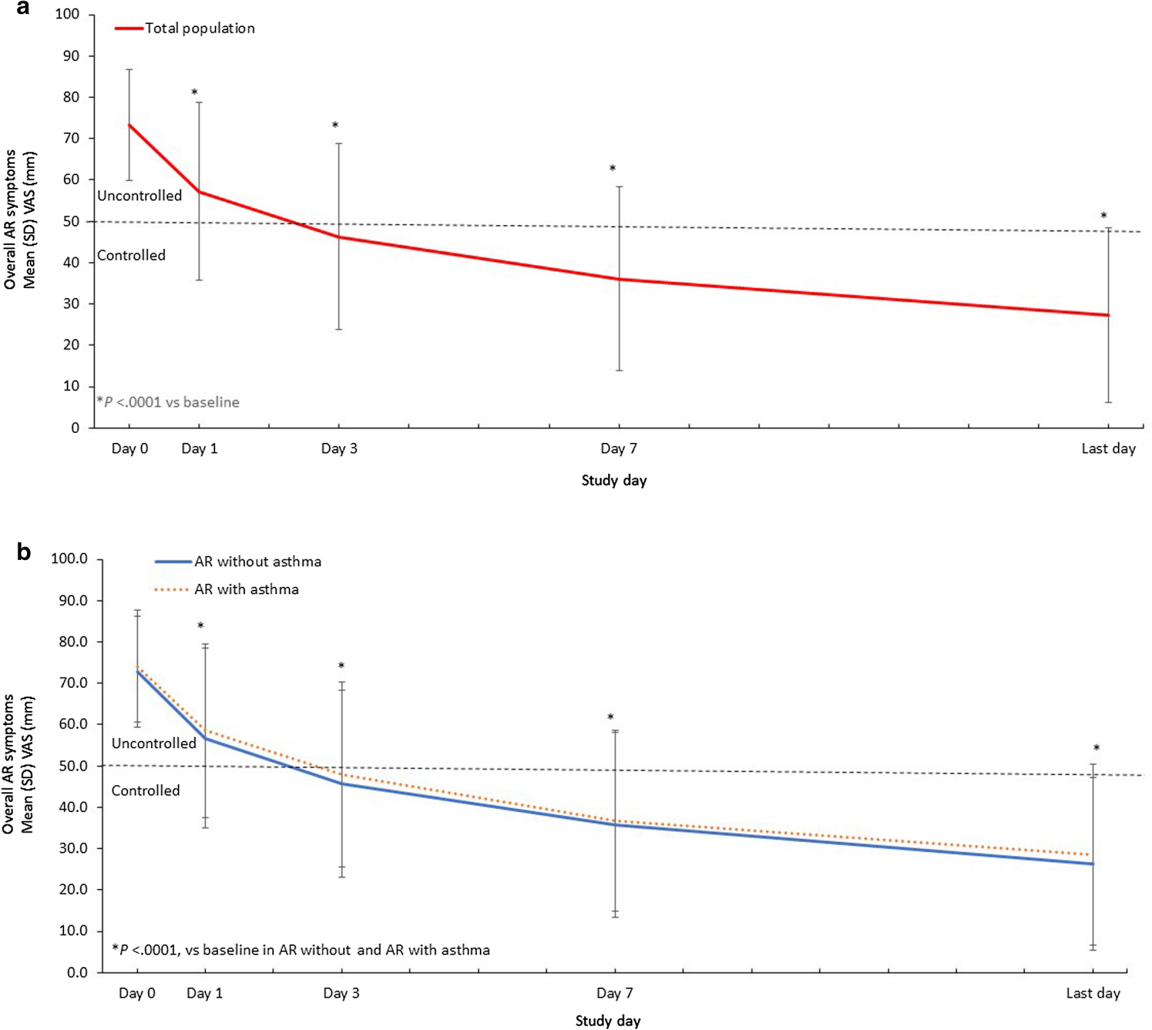
Changes in AR-VAS Scores With MP-AzeFlu Treatment. Time course of mean (SEM) VAS scores of AR symptoms from day 0 to the last trial day (**a**) in the total study population and (**b**) stratified by the presence of comorbid asthma. AR, allergic rhinitis; SD, standard deviation; SEM, standard error of the mean; VAS, visual analog scale

Treatment response was defined as an AR-VAS score < 50 mm, the cutoff that differentiates controlled AR from uncontrolled AR [20], on any 1 day. A total of 944 patients [86.6%; 95% confidence interval (CI), 84.5–88.5%) met the response criteria, including 728 patients without asthma (88.1%; 95% CI 85.8–90.2%) and 216 patients with asthma (81.8%; 95% CI 76.7–86.0%). Over the course of treatment, the mean [standard deviation (SD)] VAS decreased by 46.2 (23.3) mm from baseline to the last day (Fig. 1). The mean (SD) change in AR-VAS over the study period for patients with AR without asthma was − 46.4 (22.9) mm; for AR with comorbid asthma, it was − 45.3 (25.2) mm. For patients with and without comorbid asthma, the AR-VAS change from baseline was significant at every time point (*P* < 0.0001). Furthermore, no significant differences were observed between patients with and without asthma in AR-VAS change at any time point.

### Asthma symptom response

**Fig. 2:**
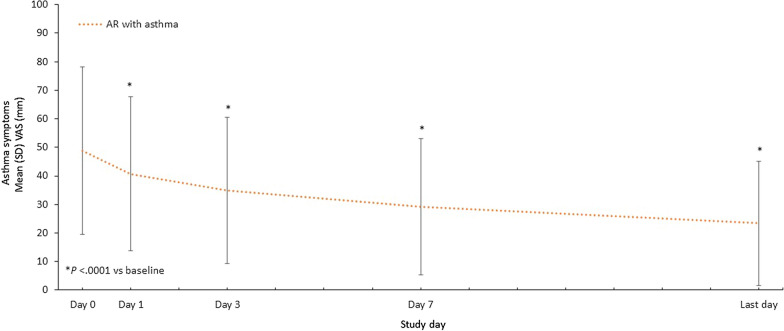
Changes in Asthma-VAS Scores With MP-AzeFlu Treatment. Time course of mean (SEM) VAS of asthma symptoms from day 0 to lthe last trial day among patients with comorbid AR and asthma. AR, allergic rhinitis; SD, standard deviation; SEM, standard error of the mean; VAS, visual analog scale

Among the subpopulation of patients with asthma, patients rated their asthma symptoms on a VAS. The mean (SD) asthma-VAS score decreased from 48.9 (29.3) mm at baseline to 24.1 (21.9) mm on the last day, resulting in a mean change of − 25.7 (26.0) mm (Fig. 2). Changes from baseline for AR symptoms and asthma symptoms were moderately correlated (Pearson correlation coefficient, 0.47; *P* < 0.0001).

Participants with asthma reported using reliever medication a mean of 5.1 times during the week before treatment. Self-reported data regarding frequency of asthma reliever medication use during the study period were available for 241 patients (85.0%). A total of 139 patients (57.6%) reported that the frequency of asthma reliever use was either considerably reduced or reduced. In addition, 93 patients (38.6%) reported no change, and 9 patients (3.7%) reported an increased frequency of asthma reliever medication use.

### Quality-of-life measurements

#### Troublesomeness of sleep

**Fig. 3:**
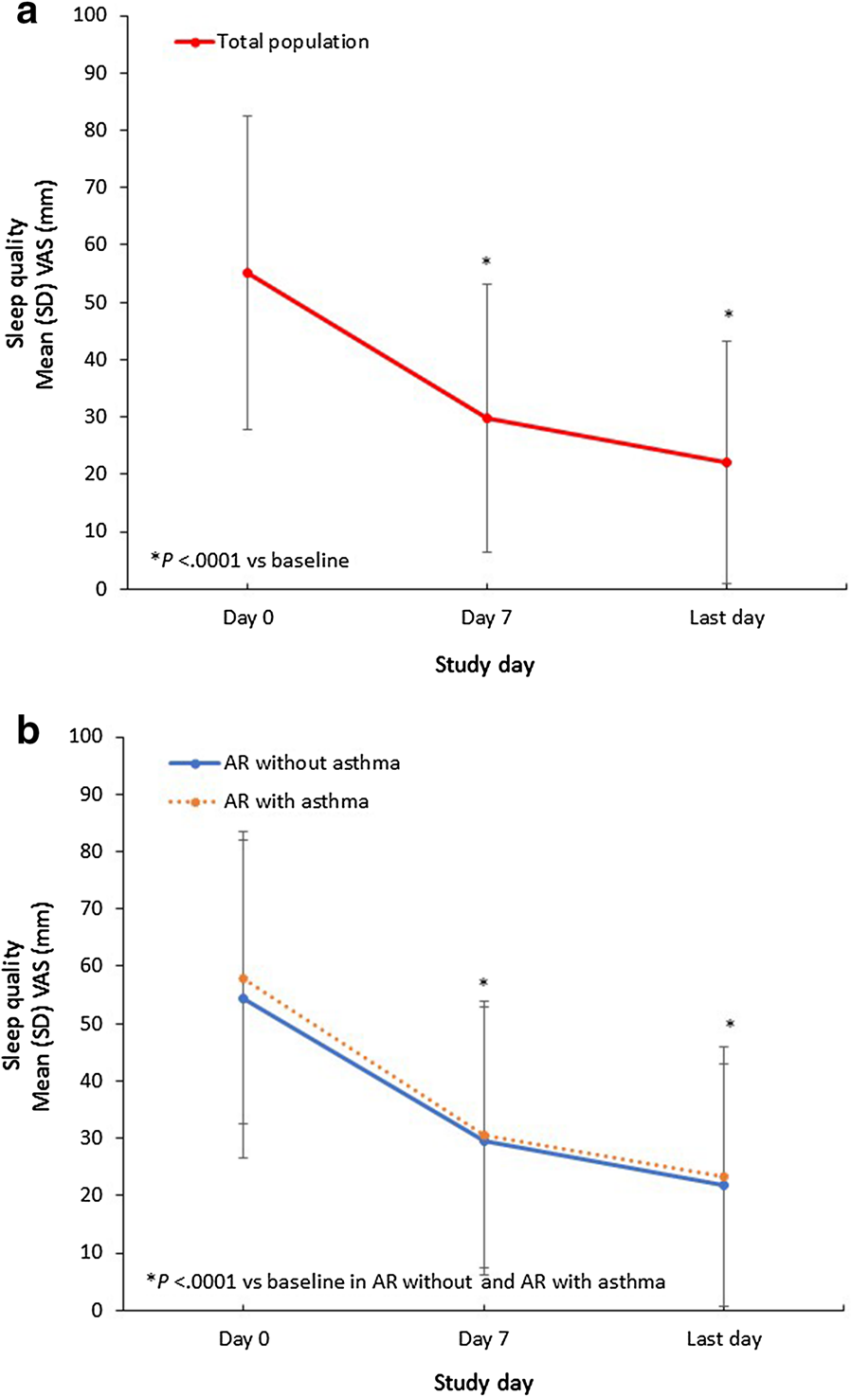
Changes in Troublesomeness of Sleep Quality VAS Scores With MP-AzeFlu Treatment. Time course of mean (SEM) VAS of change of troublesomeness of sleep quality from day 0 to the last trial day (**a**) in the total study population and (**b**) in subpopulations according to the presence of comorbid asthma. AR, allergic rhinitis; SD, standard deviation; SEM, standard error of the mean; VAS, visual analog scale

Changes in quality-of-life measurements are reported in Fig. 3 through Fig. 6. In the whole study population, mean (SD) troublesomeness with sleep quality VAS score significantly decreased by − 33.7 (28.1) mm from day 0 to the last day (*P *< 0.0001). Similarly, among the subpopulation of AR with asthma, mean (SD) troublesomeness with sleep quality VAS score decreased by 34.6 (29.1) mm from day 0 to the last day. Among the population without asthma, mean (SD) troublesomeness with sleep quality VAS score decreased by − 32.7 (28.6) mm from baseline (Fig. 3).

#### Troublesomeness of daily activities

**Fig. 4:**
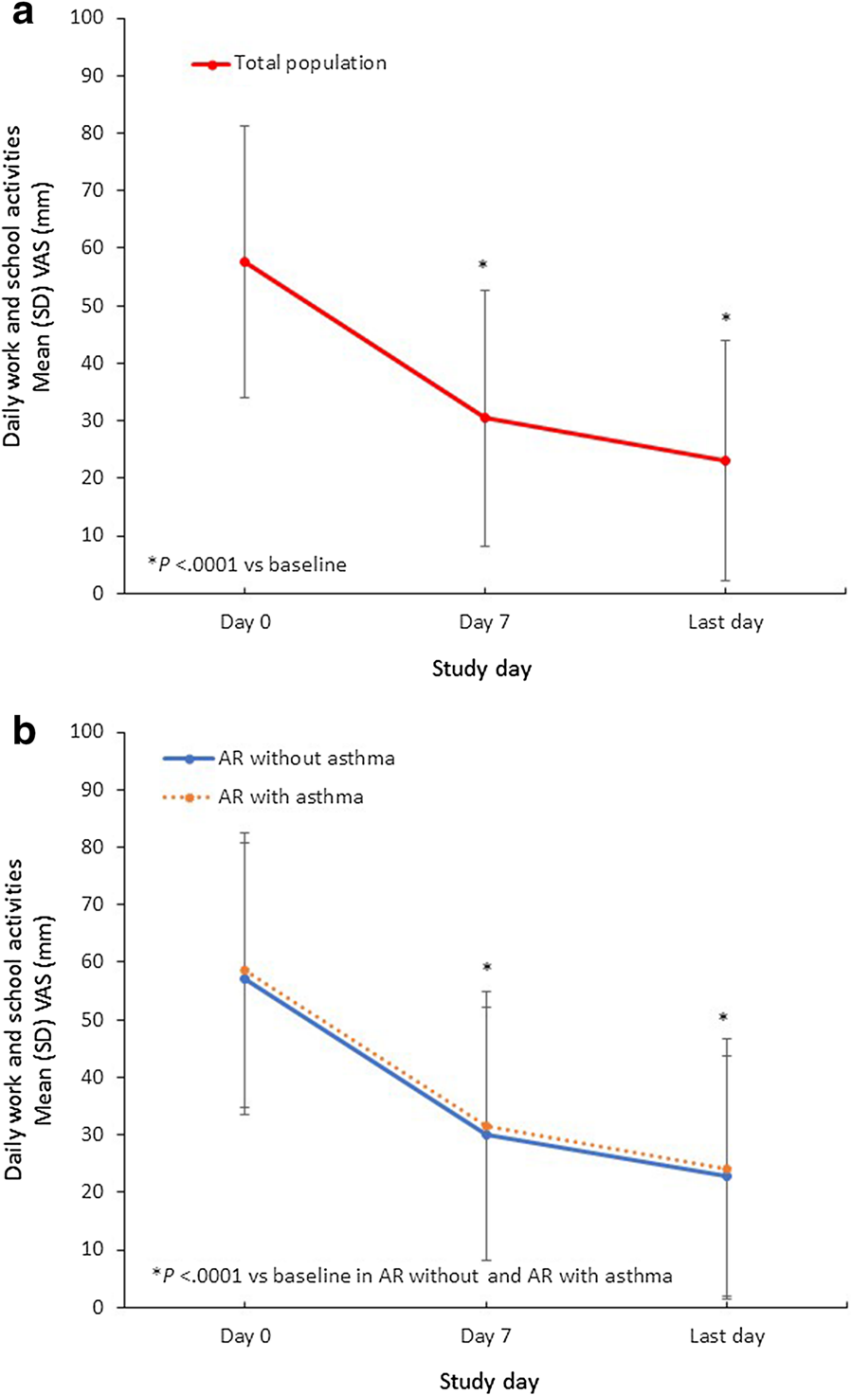
Changes in Troublesomeness of Daily Work and School Activities VAS Scores with MP-AzeFlu Treatment. Time course of mean (SEM) VAS of change of troublesomeness of daily work and school activities from day 0 to the last trial day (**a**) in the total study population and (**b**) in subpopulations according to the presence of comorbid asthma. AR, allergic rhinitis; SD, standard deviation; SEM, standard error of the mean; VAS, visual analog scale

The mean (SD) troublesomeness of daily activities at work or school VAS score significantly decreased by 35.2 (25.6) mm in the whole study population (*P* < 0.0001). In the subpopulation of AR with asthma, the mean (SD) troublesomeness of daily activities at work or school VAS score decreased by 34.3 (27.5) mm. For patients without asthma, the mean (SD) decrease from baseline in troublesomeness of daily activities was 34.7 (26.3) mm (Fig. 4).

**Fig. 5:**
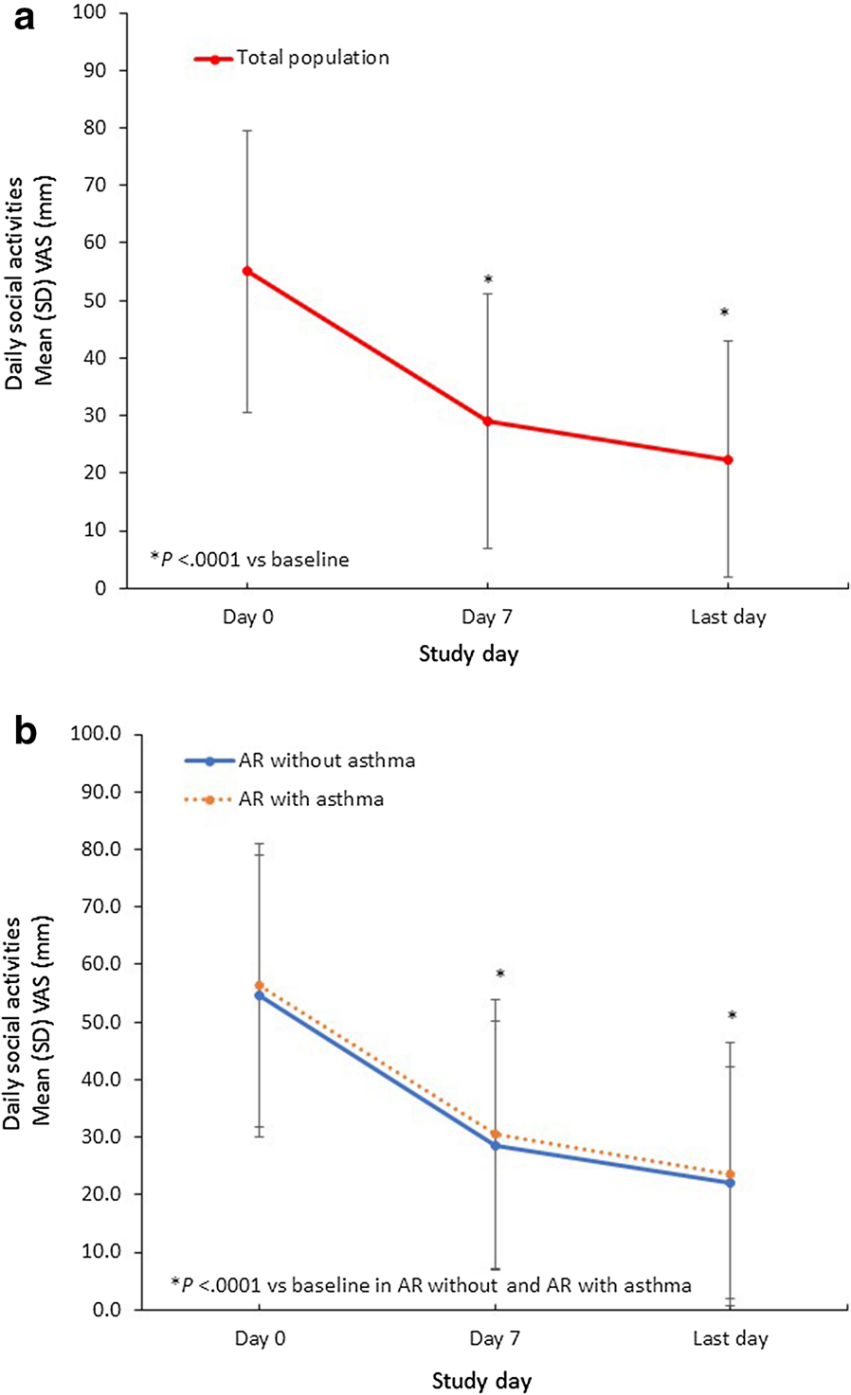
Changes in Troublesomeness of Sleep Quality VAS Scores With MP-AzeFlu Treatment. Time course of mean (SEM) VAS of change of troublesomeness of sleep quality from day 0 to the last trial day (**a**) in the total study population and (**b**) in subpopulations according to the presence of comorbid asthma. AR, allergic rhinitis; SD, standard deviation; SEM, standard error of the mean; VAS, visual analog scale

**Fig. 6:**
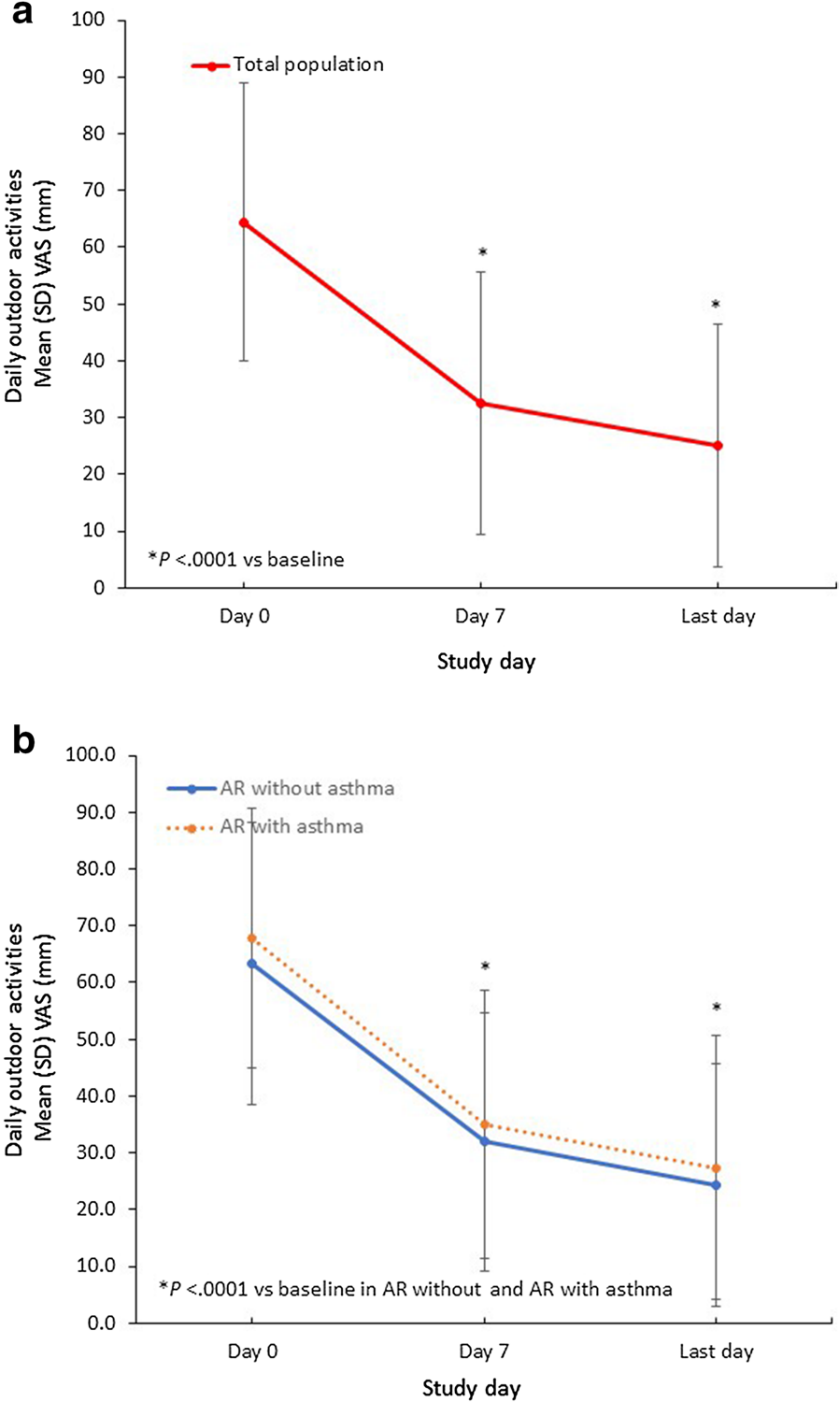
Changes in Troublesomeness of Daily Outdoor Activities VAS Scores With MP-AzeFlu Treatment. Time course of mean (SEM) VAS of change of daily outdoor activities from day 0 to the last trial day (**a**) in the total study population and (**b**) in subpopulations according to the presence of comorbid asthma. AR, allergic rhinitis; SD, standard deviation; SEM, standard error of the mean; VAS, visual analog scale

Furthermore, in the whole study population, the mean (SD) troublesomeness with daily social activities VAS score significantly decreased by 33.2 (25.8) mm from baseline to the last day (*P* < 0.0001), whereas the mean (SD) decrease in the asthma population was 32.6 (29.2) mm. Among patients with no asthma, the mean (SD) change in social activities VAS score was − 32.7 (26.1) mm (Fig. 5). Finally, mean (SD) troublesomeness with daily outdoor activities VAS scores significantly decreased by 40.0 (27.2) mm, 40.2 (30.9), and 39.2 (28.0) mm in the general study population (*P* < 0.0001), asthma subpopulation, and no asthma subpopulation, respectively (Fig. 6).

### Safety

Among the safety analysis population, 20 nonserious adverse drug reactions were reported in 14 patients (1.3%). The most frequent adverse events included epistaxis (0.4%), dysgeusia (0.3%), headache (0.2%), and dyspnea (0.2%). Among 267 patients with comorbid asthma, 5 nonserious adverse drug reactions were reported in 3 patients (1.1%), which was similar to the total population. No serious adverse drug reactions occurred.

## Discussion

This was the first multicenter, prospective, noninterventional, real-life study to evaluate the effect of AR treatment with MP-AzeFlu on asthma symptom severity and reliever medication use. A total of 24% of patients with AR in this study reported comorbid asthma, which is comparable with literature rates of 15% to 38% [1]. We showed that patients with moderate-to-severe AR and comorbid asthma treated with MP-AzeFlu had similarly improved AR symptom severity compared with patients with AR alone. Patients with and without comorbid asthma also experienced improved quality of life with MP-AzeFlu treatment. For patients with asthma, asthma symptom severity and asthma reliever medication use decreased from baseline.

In general, improvements in AR-VAS scores and quality-of-life measures were comparable for patients with and without asthma. This is particularly notable given that the subpopulation with comorbid asthma had numerically higher rates of more severe AR symptoms. Although significance testing was not performed across populations, the AR with asthma group had a higher rate of both perennial and seasonal AR and allergic sensitization to more than 5 allergens. Baseline AR-VAS scores, however, were only modestly higher in the group with asthma. These data suggest MP-AzeFlu treatment may have similar effectiveness in populations with and without asthma and with varying levels of AR severity.

VAS scores were used to assess AR symptom severity, asthma symptom severity, and quality-of-life measures in this study. Advantages of VAS measurements include a high degree of resolution, with repeat measures offering the opportunity to identify even small changes within and among individual patients and groups of patients [21]. In addition, VAS scores are good tools for measuring continuous variables, such as AR and asthma symptoms [21]. In past studies, VAS scores have been shown to correlate well with the severity of AR according to ARIA guidelines [22, 23]. A cutoff variation of 23 mm for VAS was shown to correlate well with the established cutoff of 0.5 for the Rhinoconjunctivitis Quality of Life Questionnaire [23]. Moreover, a change of 30 mm was always correlated with positive changes in quality-of-life parameters [23]. In the present study, changes in AR-VAS scores from baseline to the last day exceeded this cutoff for all endpoints in the safety population and the comorbid asthma population, suggesting meaningful changes in symptoms and quality of life.

VAS scores are not only useful in clinical practice for stratifying patients and monitoring response; they have also been used as evaluation parameters in randomized controlled trials of AR treatment. In 2 studies of AR evaluating treatment with INAH, VAS scores discriminated between placebo and treatment groups better than total symptom scores [24, 25]. In this study, the mean change in AR-VAS from baseline to the last day suggests a shift from uncontrolled to controlled AR and severe to mild AR.

Although VAS scores are less commonly used for evaluation of asthma symptoms, they have nonetheless been shown to be valid measures for predicting asthma control and lung function [26–28]. When VAS was evaluated in the morning and evening in adolescent patients with asthma, average VAS scores were significantly correlated with both asthma control (r = 0.65, *P* < 0.001) and FEV_1_ (r = − 0.38, *P* = 0.029) [26]. In a study of Japanese patients, Global Initiative for Asthma–defined control levels were discriminated by VAS score cutoff points of 1.50 cm (controlled), 4.79 cm (partly controlled), and 7.19 cm (uncontrolled) [28]. According to these cutoffs, the asthma severity VAS scores were suggestive of uncontrolled asthma at baseline, which is further supported by the use of more than 5 puffs of reliever medication on average in the week before treatment with MP-AzeFlu. With MP-AzeFlu treatment, asthma control improved by the last day to partly controlled in the majority of patients (median 20.0 mm) and to controlled in at least 25% of patients (low quartile 5.0 mm). These data are further supported by the reduced use of asthma reliever medication at study conclusion. Several studies have shown that the use of INCS can improve asthma symptoms in patients with comorbid AR through the treatment of upper airway inflammation, which indirectly decreases bronchial hyperreactivity [29, 30]. Therefore, improvement in asthma symptoms with MP-AzeFlu treatment could be attributed to the improved control of AR symptoms, decreased airway inflammation, or, most likely, a combination of the two, which is supported by the moderate correlation between AR symptom severity improvement and asthma symptom severity improvement.

The “one airway, one disease” hypothesis suggests joint management of AR and asthma leads to better control of both diseases [31, 32]. Evidence for the “one airway, one disease” hypothesis includes epidemiologic data that suggest the high frequency of comorbid asthma and AR, heritability of allergic diseases (e.g., AR, asthma, and atopic dermatitis), and the overlapping roles of inflammatory mediators in AR and asthma, which are supported by the clinical effectiveness of corticosteroids and antihistamines for both conditions. In this study, the moderate correlation between change of general AR-VAS and asthma-VAS scores lends additional credence to the “one airway, one disease” hypothesis.

Study limitations included the observational design and lack of a control group for comparative purposes. This limits comparison with previous studies, during which data were collected under different circumstances. Furthermore, we have limited data surrounding clinically relevant features of asthma, including the method by which asthma was diagnosed and current asthma medications. However, because of the multinational, noninterventional study design, information about a variety of patients’ allergy characteristics could be obtained, and comparison through a preintervention and postintervention design was recorded. Although the 2-week study period was sufficient for documenting a substantial improvement in AR and asthma symptom severity, monitoring of AR symptom control over a longer period of time would better inform long-term outcomes with MP-AzeFlu treatment.

## Conclusion

MP-AzeFlu use was associated with improved AR symptoms, asthma symptoms, and quality-of-life measures in patients with concomitant asthma. Change in overall AR symptoms and change in asthma symptoms were correlated. The results support the “one airway, one disease” therapy approach for asthma and AR management.

## Data Availability

The datasets used and/or analyzed during the current study are available from the corresponding author on reasonable request.

## Abbreviations

AR: Allergic rhinitis
ARIA: Allergic rhinitis and its impact on asthma
CI: Confidence interval
INAH: Intranasal antihistamine
INCS: Intranasal corticosteroid
SD: Standard deviation
VAS: Visual analog scale

## References

[1] Brożek JL, Bousquet J, Agache I (2017). Allergic rhinitis and its impact on asthma (ARIA) guidelines—2016 revision. J Allergy Clin Immunol..

[2] Cingi C, Gevaert P, Mösges R (2017). Multi-morbidities of allergic rhinitis in adults: European academy of allergy and clinical immunology task force report. ClinTranslat Allergy..

[3] Oka A, Matsunaga K, Kamei T (2014). Ongoing allergic rhinitis impairs asthma control by enhancing the lower airway inflammation. J Allergy Clin Immunol Pract..

[4] Clatworthy J, Price D, Ryan D, Haughney J, Horne R (2009). The value of self-report assessment of adherence, rhinitis and smoking in relation to asthma control. Prim Care Respir J..

[5] Price D, Zhang Q, Kocevar VS, Yin DD, Thomas M (2005). Effect of a concomitant diagnosis of allergic rhinitis on asthma-related health care use by adults. Clin Exp Allergy.

[6] Colas C, Brosa M, Anton E (2017). Estimate of the total costs of allergic rhinitis in specialized care based on real-world data: the FERIN Study. Allergy.

[7] Price D, Scadding G, Ryan D (2015). The hidden burden of adult allergic rhinitis: uK healthcare resource utilisation survey. Clin Translat Allergy..

[8] Price D, Kemp L, Sims E (2010). Observational study comparing intranasal mometasone furoate with oral antihistamines for rhinitis and asthma. Prim Care Respir J..

[9] Crystal-Peters J, Neslusan C, Crown WH, Torres A (2002). Treating allergic rhinitis in patients with comorbid asthma: the risk of asthma-related hospitalizations and emergency department visits. J Allergy Clin Immunol..

[10] Marple BF, Fornadley JA, Patel AA (2007). Keys to successful management of patients with allergic rhinitis: Focus on patient confidence, compliance, and satisfaction. Otolaryngol Head Neck Surg..

[11] Dymista (azelastine hydrochloride and fluticasone propionate spray, metered). In. Somerset, NJ: Meda Pharmaceuticals Inc; September 2018.

[12] Prenner BM (2016). A review of the clinical efficacy and safety of MP-AzeFlu, a novel intranasal formulation of azelastine hydrochloride and fluticasone propionate, in clinical studies conducted during different allergy seasons in the US. J Asthma Allergy..

[13] Storms W (2008). Allergic rhinitis-induced nasal congestion: its impact on sleep quality. Prim Care Respir J..

[14] Meltzer E, Ratner P, Bachert C (2013). Clinically relevant effect of a new intranasal therapy (MP29-02) in allergic rhinitis assessed by responder analysis. Int Arch Allergy Immunol.

[15] Roca-Ferrer J, Pujols L, Perez-Gonzalez M (2018). Superior effect of MP-AzeFlu than azelastine or fluticasone propionate alone on reducing inflammatory markers. Allergy Asthma Clin Immunol..

[16] Klimek L, Poletti SC, Sperl A (2017). Olfaction in patients with allergic rhinitis: an indicator of successful MP-AzeFlu therapy. Int Forum Allergy Rhinol..

[17] Kortekaas Krohn I, Callebaut I, Alpizar YA (2018). MP29-02 reduces nasal hyperreactivity and nasal mediators in patients with house dust mite-allergic rhinitis. Allergy.

[18] Harrow B, Sedaghat AR, Caldwell-Tarr A, Dufour R (2016). A Comparison of health care resource utilization and costs for patients with allergic rhinitis on single-product or free-combination therapy of intranasal steroids and intranasal antihistamines. J Manag Care Spec Pharm..

[19] Bousquet J, Khaltaev N, Cruz AA (2008). Allergic Rhinitis and its Impact on Asthma (ARIA) 2008 update (in collaboration with the World Health Organization, GA(2)LEN and AllerGen). Allergy.

[20] Bousquet J, Schunemann HJ, Hellings PW (2016). MACVIA clinical decision algorithm in adolescents and adults with allergic rhinitis. J Allergy Clin Immunol..

[21] Klimek L, Bergmann KC, Biedermann T (2017). Visual analogue scales (VAS): measuring instruments for the documentation of symptoms and therapy monitoring in cases of allergic rhinitis in everyday health care: Position Paper of the German Society of Allergology (AeDA) and the German Society of Allergy and Clinical Immunology (DGAKI), ENT Section, in collaboration with the working group on Clinical Immunology, Allergology and Environmental Medicine of the German Society of Otorhinolaryngology, Head and Neck Surgery (DGHNOKHC). Allergo J Int..

[22] Bousquet PJ, Combescure C, Neukirch F (2007). Original article: visual analog scales can assess the severity of rhinitis graded according to ARIA guidelines. Allergy.

[23] Demoly P, Bousquet PJ, Mesbah K, Bousquet J, Devillier P (2013). Visual analogue scale in patients treated for allergic rhinitis: an observational prospective study in primary care: asthma and rhinitis. Clin Exp Allergy.

[24] Bousquet J, Bachert C, Canonica GW (2010). Efficacy of desloratadine in persistent allergic rhinitis—a GA(2)LEN study. Int Arch Allergy Immunol.

[25] Bousquet J, Bachert C, Canonica GW (2009). Efficacy of desloratadine in intermittent allergic rhinitis: a GA2LEN study. Allergy.

[26] Rhee H, Belyea M, Mammen J (2017). Visual analogue scale (VAS) as a monitoring tool for daily changes in asthma symptoms in adolescents: a prospective study. Allergy Asthma Clin Immunol..

[27] Ciprandi G, Schiavetti I, Sorbello V, Ricciardolo FL (2016). Perception of asthma symptoms as assessed on the visual analog scale in subjects with asthma: a real-life study. Respir Care..

[28] Ohta K, Jean Bousquet P, Akiyama K (2013). Visual analog scale as a predictor of GINA-defined asthma control. The SACRA Study in Japan. J Asthma..

[29] Watson WT, Becker AB, Simons FE (1993). Treatment of allergic rhinitis with intranasal corticosteroids in patients with mild asthma: effect on lower airway responsiveness. J Allergy Clin Immunol..

[30] Lohia S, Schlosser RJ, Soler ZM (2013). Impact of intranasal corticosteroids on asthma outcomes in allergic rhinitis: a meta-analysis. Allergy.

[31] Grossman J (1997). One airway, one disease. Chest.

[32] Bachert C, Vignola AM, Gevaert P, Leynaert B, Van Cauwenberge P, Bousquet J (2004). Allergic rhinitis, rhinosinusitis, and asthma: one airway disease. Immunol Allergy Clin North Am..

